# RUFY3 interaction with FOXK1 promotes invasion and metastasis in colorectal cancer

**DOI:** 10.1038/s41598-017-04011-1

**Published:** 2017-06-16

**Authors:** Ruyi Xie, Jing Wang, Xuehua Liu, Liqing Wu, Hui Zhang, Weimei Tang, Yueqiao Li, Li Xiang, Ying Peng, Xiaoting Huang, Yang Bai, Guangnan Liu, Aimin Li, Yadong Wang, Ye Chen, Yuexin Ren, Guoxin Li, Wei Gong, Side Liu, Jide Wang

**Affiliations:** 1 Guangdong Provincial Key Laboratory of Gastroenterology, Department of Gastroenterology, Nanfang Hospital, Southern Medical University, Guangzhou, 510515 China; 2 Department of Gastroenterology, The First People’s Hospital of Shunde, Foshan, 528300 China; 3 Department of Gastroenterology, Hexian Memorial Affiliated Hospital of Southern Medical University, Guangzhou, 511400 China; 4 Department of Gastroenterology, Longgang District People’s Hospital, Shenzhen, 518172 China; 5 Department of General Surgery, Nanfang Hospital, Southern Medical University, Guangzhou, 510515 China

## Abstract

RUFY3 is highly expressed in brain tissue and has a role in neuronal development. Transcriptional factor FOXK1 is involved in cell growth and metabolism. We knew that RUFY3 or FOXK1 has been correlated with the malignant of tumor cells. However, the role of these molecules in colorectal cancer (CRC) progression remains unknown. We investigated the protein expression levels by Western blot, immunofluorescence and immunohistochemistry analyses. The migration and invasive abilities of CRC cells were assessed using shRNA-mediated inhibition *in vitro* and *in vivo*. We showed that RUFY3 expression was up-regulated in CRC compared with its expression in a normal human colon cell line (FHC). RUFY3 suppression inhibited anchorage independent cell tumorigenesis. RUFY3 induced elevated expression of eight major oncogenes. Moreover, RUFY3 physically interacts with FOXK1 in CRC. A positive correlation was observed between the expression patterns of RUFY3 and FOXK1. Furthermore, RUFY3 and FOXK1 expression were correlated with tumor progression and represented significant predictors of overall survival in CRC patients. SiRNA-mediated repression of FOXK1 in RUFY3-overexpressing cells reversed the epithelial-mesenchymal transition (EMT) and metastatic phenotypes. *In vivo*, FOXK1 promoted RUFY3-mediated metastasis via orthotopic implantation. These findings suggest that the RUFY3-FOXK1 axis might promote the development and progression of human CRC.

## Introduction

The RUFY proteins, or the RUN and FYVE domain-containing protein family, contain an amino-terminal RUN domain and a carboxyl-terminal FYVE domain^1, 2^. Sequence and genome analysis has revealed that the RUFY family consists of 4 protein members, including RUFY1/Rabip4, RUFY2, RUFY3/SINGAR1 and RUFY4^3–6^. RUFY proteins are predominantly localized in the early endosomes and regulate diverse cellular processes, including cell migration, actin cytoskeleton dynamics^7^, lipid modification^8^, membrane trafficking^9^ and cell signaling^10^. The overexpression of RUFY1 leads to constitutive presentation of αv integrins at the leading edge, whereas inhibition of RUFY1 expression is associated with impaired presentation of αv integrins at the cell periphery, indicating that RUFY1 is a potential regulator of integrin trafficking during neoplastic cell migration and invasion^11^.

RUFY3, also known as RIPX, is diffusely localized in hippocampal neurons and accumulated in the growth cones and axons^12^. RUFY3 also contains the RUN domain and seems to play important roles in multiple Ras-like GTPase signaling pathways^13^. Rab5 engages in a GTP-dependent interaction with RUFY3. RUFY3 can bind to the active Rab5 and weakly associates with Rap2^13^. A recently published report indicates that RUFY3 overexpression leads to the formation of F-actin-enriched protrusive structures at the cell periphery. P21-activated kinase-1 (PAK1) interacts with RUFY3, resulting in RUFY3-induced gastric cancer cell migration^14^. However, its pathophysiologic role and relevance to colorectal cancer (CRC) metastasis remain unexplored.

Forkhead box k1 (FOXK1) is a transcription factor that belongs to the forkhead family of the winged-helix DNA-binding domain and N-terminal and C-terminal transcriptional domains^15–17^. Recent progress has highlighted the significance of FOXK1 in tumor progression. Wang *et al*.^18^ found that FOXK1 and FOXK2 positively regulate Wnt/β-catenin signaling by translocating DVL into the nucleus. FOXK1 is an oncogenic factor in gastric cancer and CRC^19–22^. Protein-protein interaction analysis using the STRING database showed that RUFY3 and FOXK1 may have been some co-precipitation reactions. However, the effects of the interaction between RUFY3 and FOXK1 on EMT and tumor invasion/metastasis for CRC remain unknown.

In this study, we showed that RUFY3 physically interacts with FOXK1 in CRC. Moreover, FOXK1 short interfering RNA (siRNA) reversed these effects in cells that overexpressed RUFY3. These results indicated that tumor progression and metastasis were promoted by a key signaling pathway involving a RUFY3-FOXK1 axis.

## Results

### RUFY3 facilitates the malignant biological behavior of CRC cells

**Figure 1:**
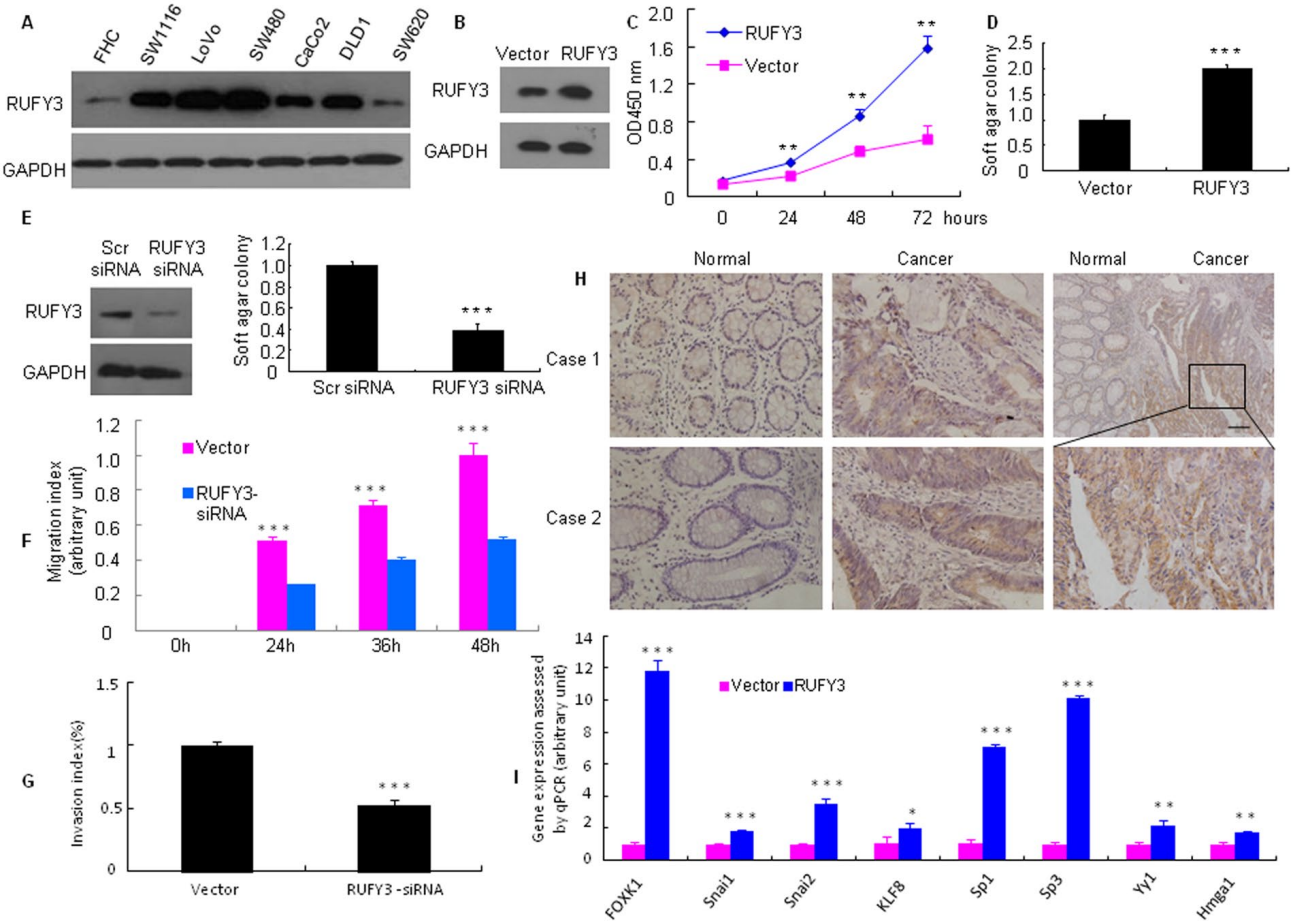
RUFY3 is overexpressed in human CRC cells. (**A**) RUFY3 expression was detected by western blot with GAPDH used as the internal control. (**B**,**C**) The proliferation of pooled stable RUFY3 transfectants and vectors was evaluated using a WST-1 assay. **P < 0.01. (**D**,**E**) Cells were transfected with RUFY3-siRNA or scr-siRNA (scramble siRNA) for 48 h, which was followed by western blot analysis. Vector and pooled stable RUFY3 transfectants, Scr siRNA and RUFY3 siRNA in LoVo cells and their corresponding control cells were plated in tissue culture dishes. Colonies containing >50 cells were considered viable, and the results are presented as the means ± SD. ***P < 0.001. (**F**) Knockdown of RUFY3 led to a significantly slower migration at 24, 36, and 48 h after cells were infected with RUFY3 siRNA. ***P < 0.001. (**G**) Inhibition of RUFY3 led to a reduced invasive ability of LoVo cells. ***P < 0.001. (**H**) RUFY3 expression in normal and malignant human colorectal tissues was detected by IHC. Scale bars, 100 μm. (**I**) Expression changes of 8 major oncogenes in pooled stable transfectants of Vector or RUFY3, as detected by qRT-PCR in LoVo cells. *P < 0.05, **P < 0.01, and ***P < 0.001. All experiments were repeated at least twice. The full-length blots/gels are presented in Supplementary Figure 3.

We first showed that RUFY3 protein expression was significantly increased in the SW1116, LoVo, SW480, CoCo2, DLD1 and SW620 cancer cell lines compared with the expression in a normal human colon cell line (FHC) (Fig. 1A).

To assess the effect of constitutive expression of RUFY3 on the growth characteristics of CRC cells *in vitro*, we established pooled stable transfectants of RUFY3 and vector control, pENTER in LoVo cells (as confirmed by western blot analysis, Fig. 1B). The WST-1 assay revealed that cell proliferation was significantly higher than that in the control vector cells (Fig. 1C).

Anchorage-independent growth is a pivotal characteristic of malignant transformation. Therefore, a soft-agar assay was performed to identify the function of RUFY3. As expected, 12 days later, the cell clone number of RUFY3 overexpression was significantly increased (Fig. 1D and Supplementary Figure 1A), while RNAi-mediated repression of RUFY3 decreased, compared with that of the control in LoVo cells (Fig. 1E and Supplementary Figure 1B).

Elevated cell migration and invasion are associated with increase in the metastatic potential of cancer cells. This may be independent of cell proliferation rates. Therefore, we studied the effect of RUFY3 downregulation on the migration and invasion of CRC cells. Cell migration was determined by wound healing assay. As shown in Fig. 1F and Supplementary Figure 2A, RUFY3 downregulation significantly suppressed the migration of LoVo cells. The migration index of RUFY3 knockdown cells was decreased by 49.1%, 42.2%, and 48.0% at 24, 36 and 48 h, respectively (Fig. 1F). To examine the cell invasion activity *in vitro*, we used transwell inserts coated with matrigel matrix. After RUFY3 knockdown, the invasiveness of LoVo cells was decreased by 47.7% as compared with the control cells (Fig. 1G and Supplementary Figure 2B).

Next, we detected RUFY3 expression *in situ* by immunohistochemistry (IHC) in tissue specimens collected from colons with noncancerous or cancerous diseases. We found that RUFY3-positive signals were present of cancer cells (Fig. 1H). In contrast, normal colon tissues either did not express or weakly expressed RUFY3 protein. These findings demonstrated that RUFY3 was overexpressed in CRC.

Finally, we screened for the potential target genes of RUFY3 by examining the expression changes of 8 major oncogenes after ectopic RUFY3 expression in LoVo cells. The mRNA expression levels of FOXK1, SNAI1, SNAI2, KLF8, Sp1, ZEB1, YY1 and HMGA1 were up-regulated in pooled stable RUFY3 transfectants (Fig. 1I).

These results indicate that the RUFY3 facilitates the malignant biological behavior of CRC cells.

### RUFY3 physically interacts with FOXK1 in CRC

**Figure 2:**
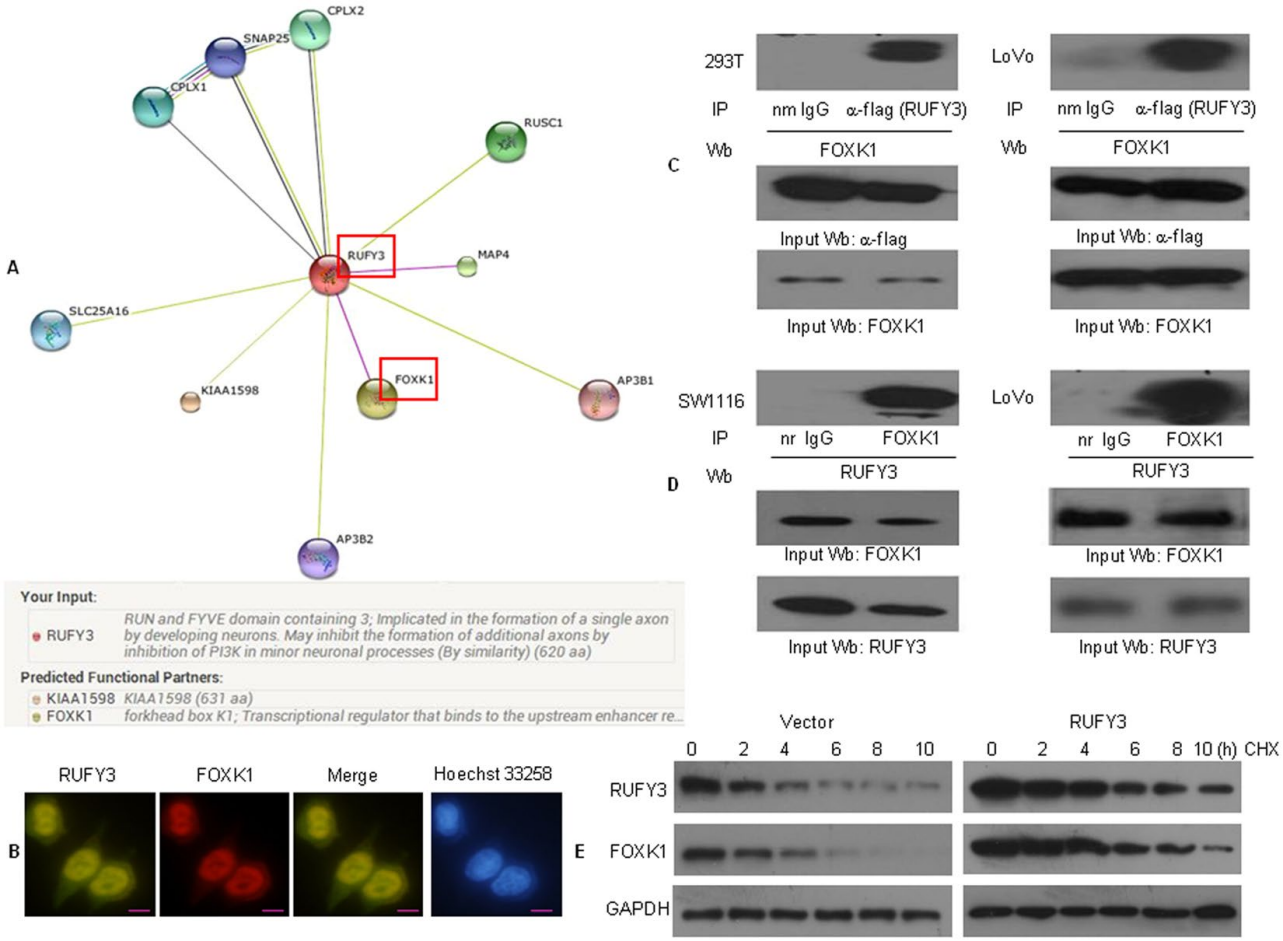
RUFY3 physically interacts with FOXK1 in CRC. (**A**) Potential RUFY3-binding partners were predicted using a STRING database. Red boxes represent protein-protein interactions. (**B**) Double staining of RUFY3 and FOXK1 in LoVo cells by indirect immunofluorescence, with the nuclei counterstained Hoechst 33258. Scale bars represent 20 μm. (**C**) Flag-tagged RUFY3 plasmid was transfected into HEK293 and LoVo cells. Immunoprecipitation was performed with anti-flag antibody, and pre-immune normal mouse immunoglobulin G (nm IgG) was used as a control. Western blot analysis was performed with an anti-FOXK1 antibody. IP: immunoprecipitation; Wb: Western blot. (**D**) Cell lysates of SW1116 and LoVo cells were immmunoprecipitated by anti-FOXK1 antibody or the control antibody, normal rabbit immunoglobulin G (nr IgG). Western blotting was performed with anti-RUFY3 antibody. All experiments were repeated 2 to 3 times with similar findings. (**E**) Regulation of FOXK1 protein stability by RUFY3. At the start of the experiment, 50 mg/ml cycloheximide (CHX) was added to vector LoVo cells and to cells expressing RUFY3. Cells were harvested and lysates were prepared at the indicated times after the addition of cycloheximide. Western blotting was performed. The full-length blots/gels are presented in Supplementary Figure 5.

We determined that RUFY3 protein was predicted to interact with FOXK1 protein using the STRING database (Fig. 2A). Thus, we sought to clarify the cellular distribution of the two proteins. A merged signal indicates the co-localization of the two proteins via a two-color immunofluorescence assay (Fig. 2B). In addition, we confirmed the interaction between RUFY3 and FOXK1 proteins using the transient transfection of full-length, flag-tagged RUFY3. Co-immunoprecipitation showed that FOXK1 could be co-precipitated with flag-tagged-RUFY3 in human embryonic kidney 293 T cells (HEK 293 T cells) and LoVo cells (Fig. 2C). To further verify that the RUFY3-FOXK1 interaction occurs with endogenous RUFY3, whole cell lysates from SW1116 and LoVo cells were prepared for immunoprecipitation. Indeed, endogenous RUFY3 was also capable of binding to FOXK1 (Fig. 2D).

To determine whether enhanced protein stability accounts for the enhanced levels of FOXK1 found in cells expressing RUFY3, we treated vector and LoVo cells expressing RUFY3 with cycloheximide and prepared extracts after different times (Fig. 2E and Supplementary Figure 4). Protein level of FOXK1 decreased in vector cells with a half‐life of approximately 4 h, demonstrating that protein was subject to proteolysis. In contrast, protein was essentially stable over the entire time period in cells expressing RUFY3 (estimated half‐life: FOXK1: 8 h). Therefore, expression of RUFY3 stabilized FOXK1 in LoVo cells.

Collectively, these findings confirmed that RUFY3 can physically interact with and stabilize FOXK1.

### Positive correlation between high RUFY3 and FOXK1 levels in CRC

**Figure 3:**
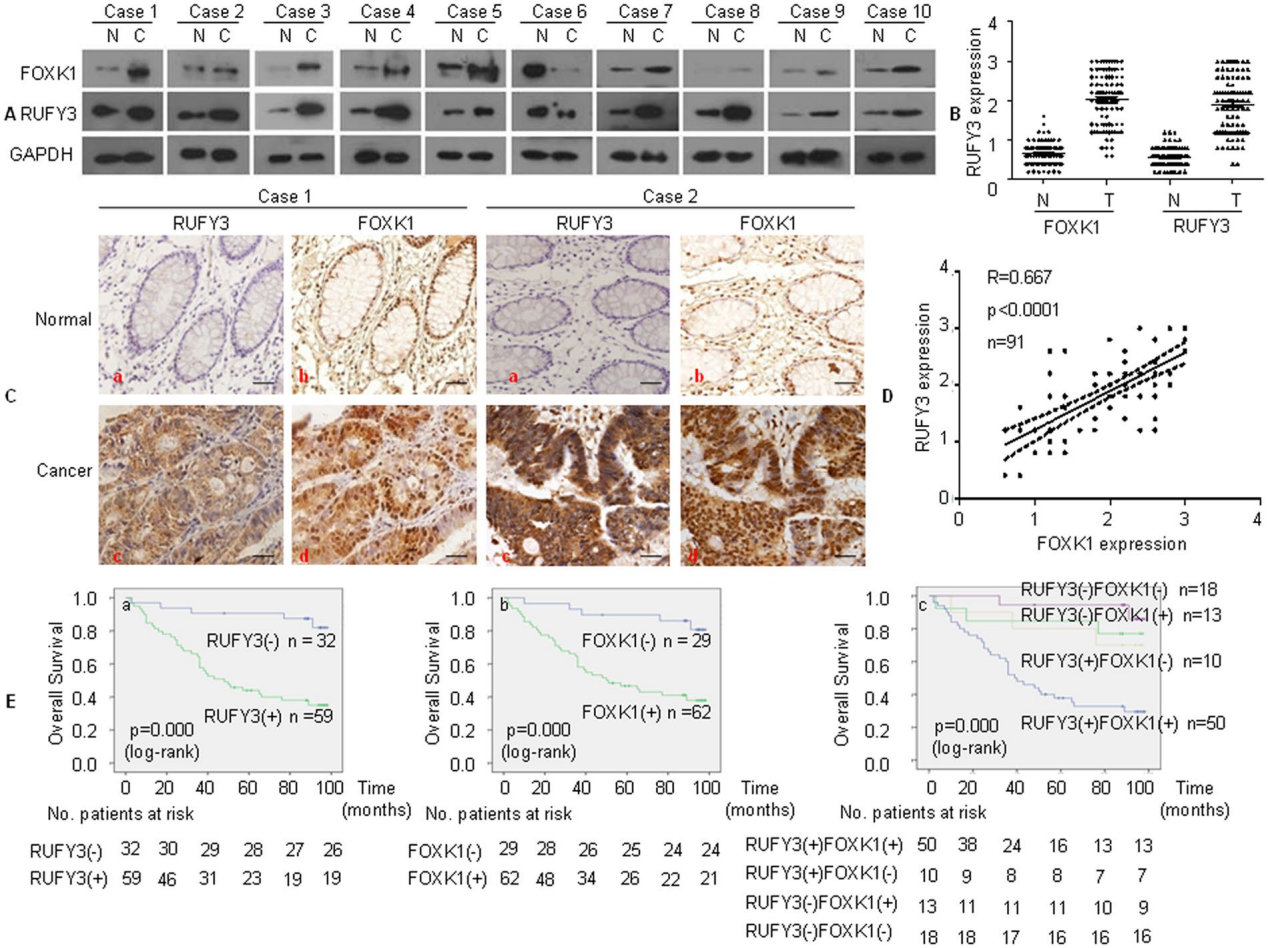
RUFY3 expression positively correlates with FOXK1 expression in human CRC. (**A**) Western blot examination of RUFY3 and FOXK1 protein expression in ten freshly collected CRC biopsies. (**B**) Average scores of the two proteins in normal and cancerous colon tissues. (**C**) RUFY3 (a, c) and FOXK1 (b, d) expression in normal or cancerous colon tissue specimens was detected by immunohistochemistry. Scale bars represent 20 μm. (**D**) Positive staining for RUFY3 and FOXK1 was quantified, and their correlation was analyzed using Spearman’s correlation method. (**E**) Kaplan-Meier overall survival analysis of CRC patients. Survival analysis was performed according to the expression status of RUFY3 (a), FOXK1 (b), and the combined expression status of RUFY3 and FOXK1 (c), respectively. The full-length blots/gels are presented in Supplementary Figure 7.

Here, we investigated whether RUFY3 and FOXK1 expression are correlated in CRC. We analyzed the expression of RUFY3 and FOXK1 in ten freshly collected CRC biopsies. Western blot analyses indicated that both RUFY3 and FOXK1 were significantly upregulated in the nine of ten examined tumor samples compared with the paired adjacent noncancerous tissues from the same patients (Fig. 3A).

Then, we found that the up-regulation of RUFY3 increased FOXK1 expression, whereas down-regulation of FOXK1 in RUFY3-overexpressing cells using siRNA decreased FOXK1 expression in two CRC lines (Supplementary Figure 6A).

To validate our findings *in vivo*, we investigated the correlation between RUFY3 and FOXK1 expression in 91 pairs of adjacent normal colon mucosal tissues and cancer tissues (Fig. 3B). Both proteins were highly expressed by cancer cells, but they were either not expressed or were expressed at extremely low levels in normal tissues (Fig. 3C). Moreover, we demonstrated that the RUFY3 and FOXK1 expression levels in CRC were concordant. After calculating the regression coefficient between the expression scores of RUFY3 and FOXK1, we found that there was a significant linear regression between RUFY3 and FOXK1 in primary CRC (Fig. 3D).

These results suggest that there is a strong positive correlation between the RUFY3 and FOXK1 expression levels in CRC.

### Co-expression of RUFY3 and FOXK1 is associated with adverse prognosis in primary CRC

To explore the clinical relevance of RUFY3 and FOXK1 expression, we analyzed the clinicopathological features in CRC. Among these 91 patients, RUFY3 or FOXK1 expression in tumour samples was found to be significantly correlated with tumour differentiation, AJCC, lymph node metastasis, TNM stage and serosel invasion; however, it was not correlated with gender, age, location or tumor size (Supplementary Table 2).

To analyze the correlation between RUFY3 (Fig. 3E-a) or FOXK1 (Fig. 3E-b) expression and the prognosis of CRC patients, Kaplan-Meier survival curves were generated. A high positive expression of each protein was correlated with poor outcome (Fig. 3E-a and b). Double positive cases that expressed both proteins showed the worst prognosis (Fig. 3E-c). Representative IHC images for tissues are shown in Supplementary Figure 6B. Thus, co-expression of RUFY3 and FOXK1 correlates with a poor prognosis in human CRC.

### RUFY3 cooperates with FOXK1 to promote migration and invasion of CRC cells

**Figure 4:**
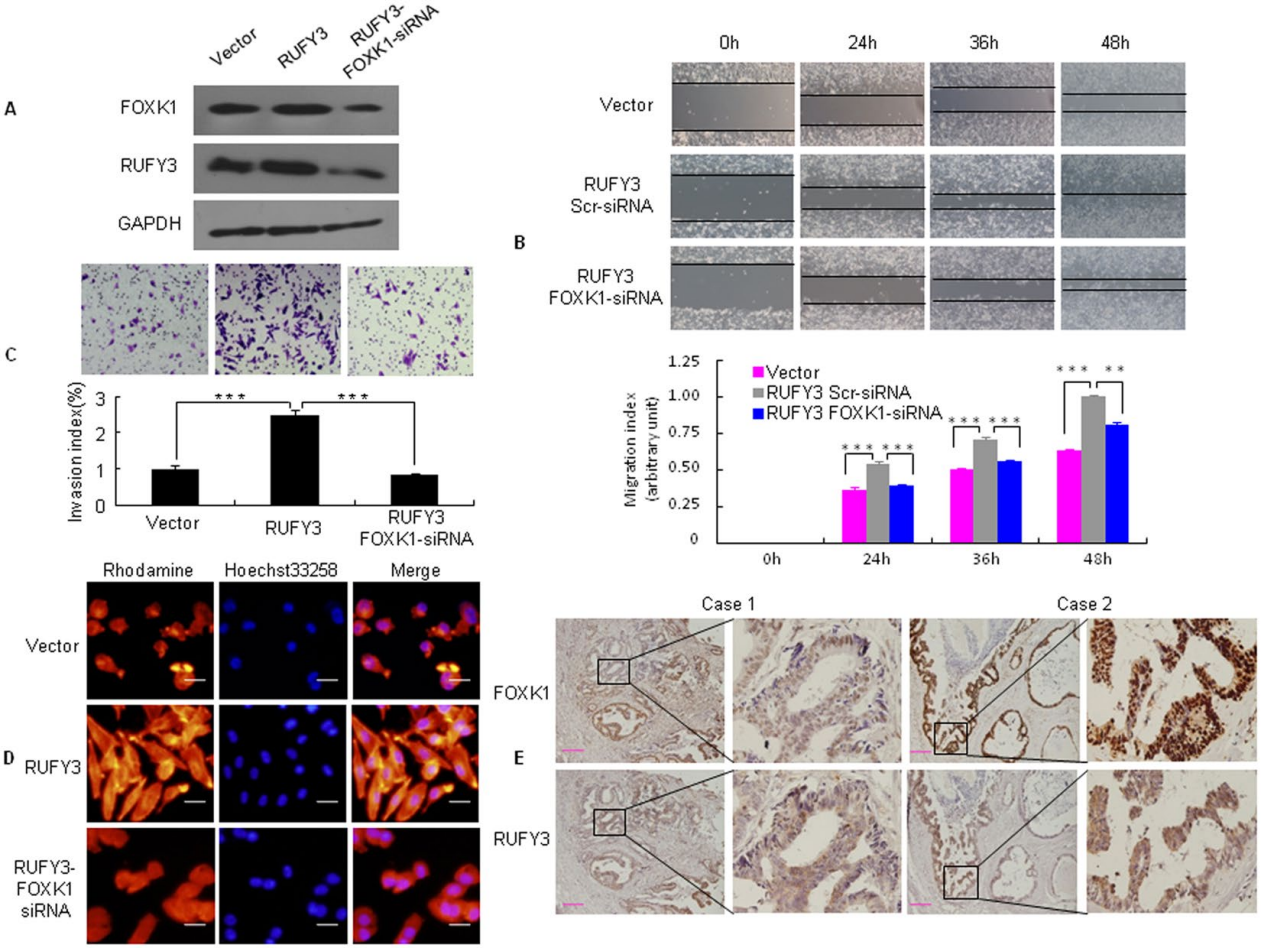
RUFY3 cooperates with FOXK1 to promote the migration and invasion of CRC cells. (**A**) Stable transfectants with vector, pooled stable transfectants with RUFY3 transfected with FOXK1 siRNA, and RUFY3 expression were detected by western blot. (**B**) For the wound healing experiments, cells were analyzed with live-cell microscopy. Original magnification, 10x. ***P < 0.001. (**C**) Invasive potential of LoVo/Vector, LoVo/RUFY3, and LoVo/RUFY3-FOXK1 siRNA. ***P < 0.001. (**D**) LoVo cells stained with rhodamine-phallotoxin for 48 h to identify F-actin filaments were visualized under fluorescent microscopy. (**E**) Representative IHC images are shown for RUFY3 and FOXK1 expression in lymph node metastatic cancer tissues. Scale bars represent 20 μm in **D** and 100 μm in **E**. All experiments were repeated three times with similar findings. The full-length blots/gels are presented in Supplementary Figure 8.

We and others have implicated knockdown of FOXK1 exerts anti-oncogenic effect in tumor cells *in vitro* and *in vivo* ^19, 20^. To investigate whether FOXK1 regulates RUFY3 in CRC, we downregulated FOXK1 in RUFY3-overexpressing cells using siRNA. We confirmed this effect by western blot in LoVo cells (Fig. 4A). Knockdown of FOXK1 in RUFY3-overexpressing cells led to a decrease in the migratory potential of RUFY3-overexpressing cells *in vitro* in a wound healing assay (Fig. 4B). Similarly, FOXK1 downregulation in RUFY3-overexpressing cells decreased the invasion potential of RUFY3-overexpressing cells by 34.5% (Fig. 4C).

A recently published report indicated that RUFY3 overexpression leads to the formation of F-actin-enriched protrusive structures at the cell periphery in primary cortical neural progenitor cells^14^. We then stained F-actin using phalloidin. Compared with the vector-expressing cells, RUFY3-overexpressing cells showed F-actin staining throughout the cytoplasm and protrusion of the leading edge (Fig. 4D). Conversely, FOXK1 knockdown in RUFY3-overexpressing cells led to weak and aggregated F-actin filaments that are involved in cancer cell invasion and metastasis.

To validate our findings *in vivo*, we detected RUFY3 and FOXK1 in serial sections of lymph node metastatic cancer tissues from two patients. RUFY3 and FOXK1 were expressed at high and intermediate levels, respectively, in the cytoplasm and nucleus of cancer cells (Fig. 4E).

Together, these data suggest that co-expression of RUFY3 and FOXK1 may be involved in CRC cell invasion and metastasis.

### RUFY3 and FOXK1 mutually promote EMT-like phenotypes in CRC cells

**Figure 5:**
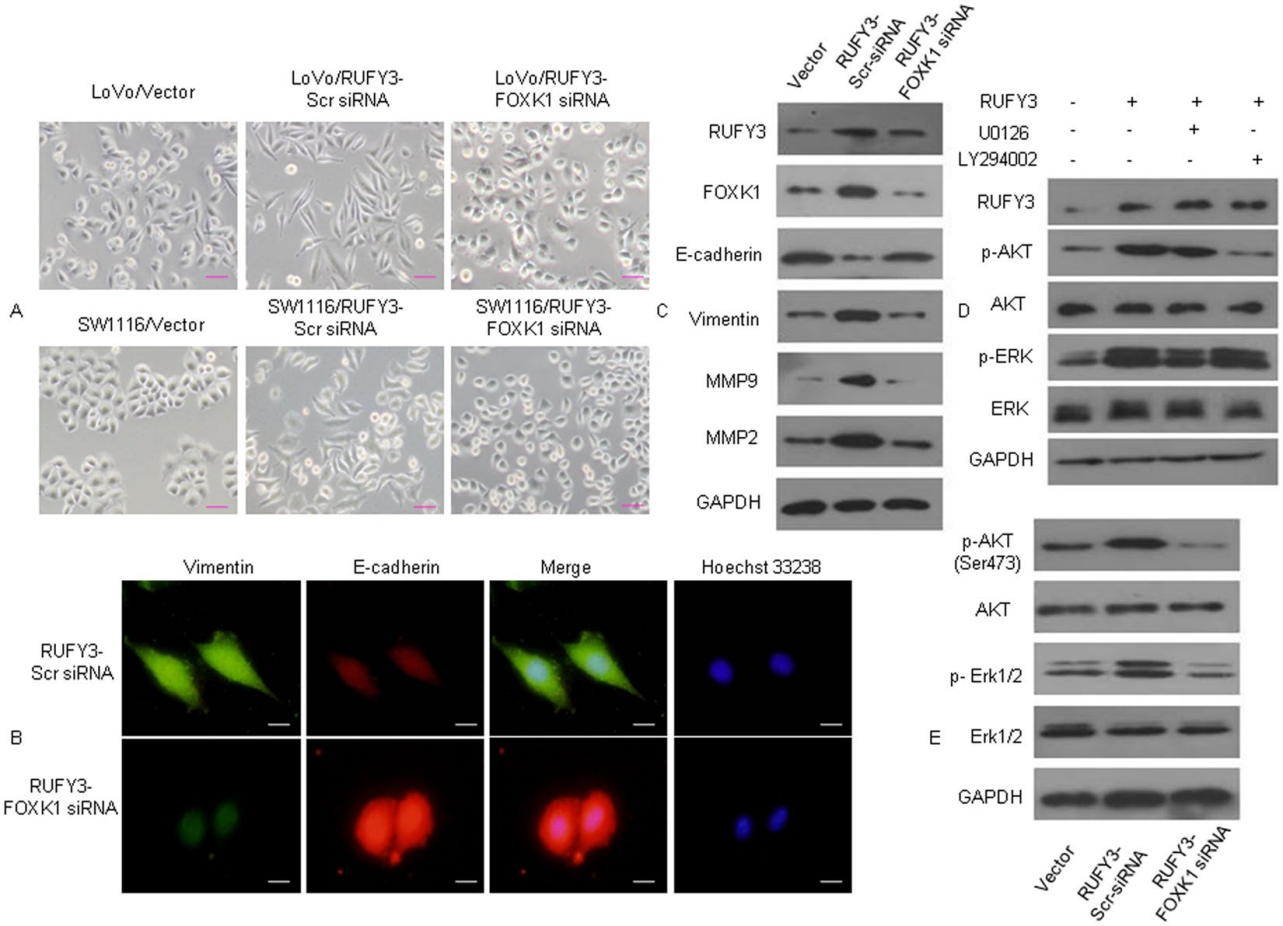
FOXK1 regulates EMT *in vitro*. (**A**) Morphology of pooled stable transfectants of Vector, RUFY3, and RUFY3-FOXK1 siRNA, as visualized under a phase-contrast microscope. (**B**) Immunofluorescence and microscopic visualization of E-cadherin (red) and vimentin (green) staining in RUFY3-scr-siRNA and RUFY3-FOXK1-siRNA cells. (**C**) The EMT biomarkers, including E-cadherin, vimentin, MMP2 and MMP9, were detected by western blot. (**D**) Western blot analyses of pooled stable transfectants with vector and RUFY3, examining p-ERK and p-Akt expression in LoVo cells treated with U0126 or LY294002. (**E**) Decreased FOXK1 expression inhibited the AKT/ERK/EMT signaling pathway, as detected using western blot analysis. Scale bars represent 50 μm in (**A**) and 20 μm in (**B**). The results were reproduced in three independent experiments. The full-length blots/gels are presented in Supplementary Figure 9.

EMT is the key process that drives cancer metastasis. The loss of E-cadherin expression and gain of vimentin expression are the most important molecular markers of EMT^23–26^. Recently, we showed that FOXK1 induces EMT in CRC cells^19^. We then examined the morphologic features of these cells. The pooled stable vector transfectants displayed a round or flat morphology with short cytoplasmic processes. However, the RUFY3 transfectants exhibited a spindle-like, fibroblastic morphology, which is one of the main EMT characteristics. Long or dendritic-like cytoplasmic processes were visible under a phase-contrast microscope. Conversely, FOXK1 knockdown in RUFY3 overexpressing cells led to EMT reversion (Fig. 5A). Immunofluorescence staining of E-cadherin and vimentin confirmed the EMT-associated shift in marker expression (Fig. 5B). Moreover, the expression of a typical EMT epithelial marker, E-cadherin, was up-regulated after the knockdown of FOXK1 in RUFY3-overexpressing cells. In contrast, the mesenchymal markers MMP-2, MMP-9 and Vimentin were downregulated (Fig. 5C).

Next, we performed western blot analysis to elucidate the phosphorylation statuses of AKT and ERK, which are involved in EMT signaling. Ectopic expression of RUFY3 led to high phosphorylation levels of p-Akt and p-ERK compared with that observed in vector cells. RUFY3-overexpressing cells were treated with the ERK inhibitor U0126 or the PI3K/Akt inhibitor LY294002 for 2 days. U0126 treatment inhibited activation of p-ERK, but not p-Akt, whereas treatment with LY294002 had the opposite effect (Fig. 5D).

Finally, the AKT and ERK1/2 phosphorylation levels were downregulated after FOXK1 knockdown in RUFY3-overexpressing cells compared with FOXK-overexpressing cells, and the total AKT and ERK1/2 protein levels were unaltered (Fig. 5E).

Together, these data suggest that the RUFY3-FOXK1 axis promotes EMT-like phenotypes in CRC cells.

### RUFY3 synergizes with FOXK1 to promote tumor metastasis *in vivo*

**Figure 6:**
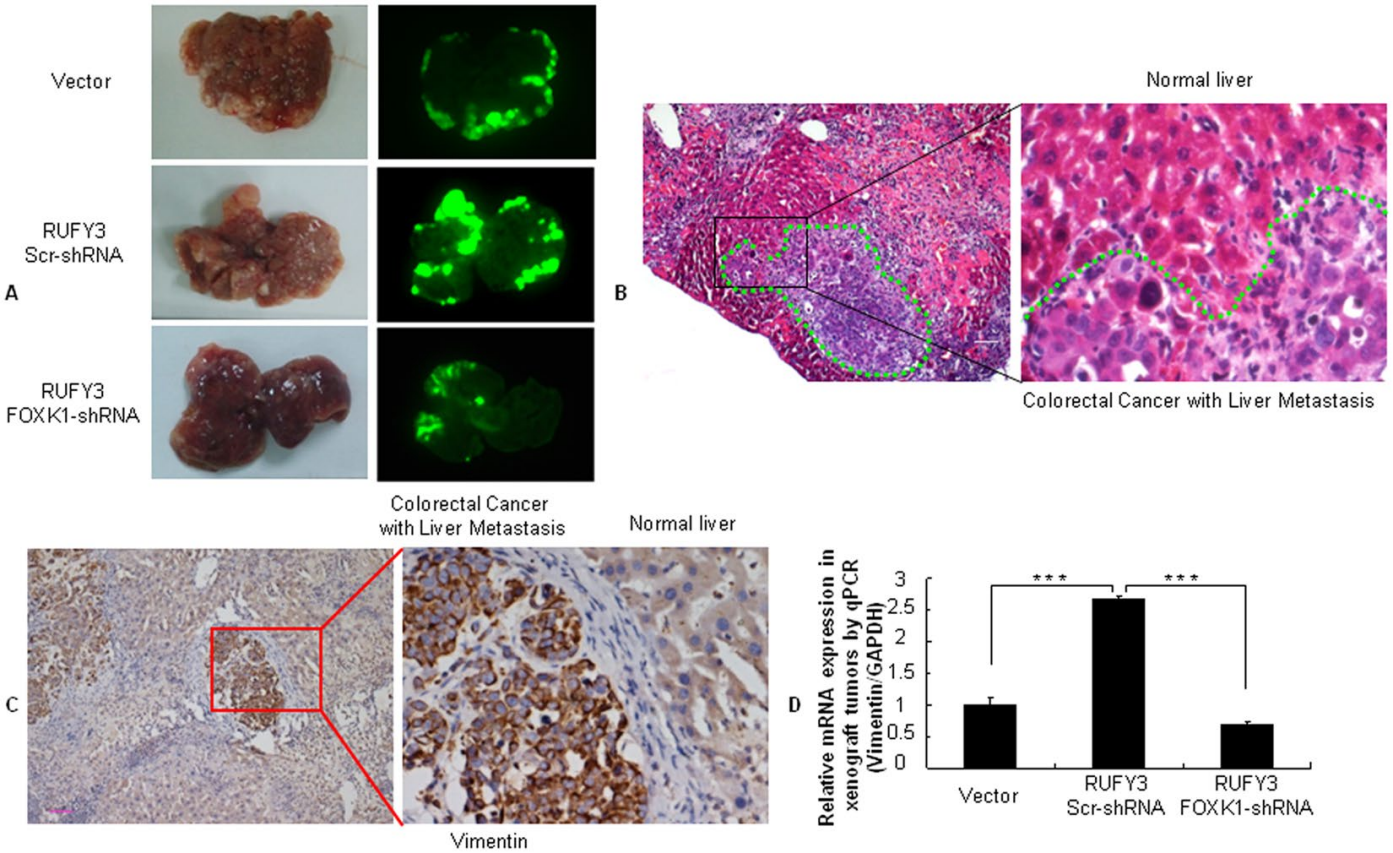
RUFY3 synergizes with FOXK1 to promote tumor metastasis *in vivo*. (**A**) External whole-body fluorescence images of the liver by injection of LoVo/pEGFP-N1(Vector), LoVo/pEGFP-RUFY3-scr-shRNA and LoVo/pEGFP-RUFY3- FOXK1-shRNA were obtained 30 days after spleen injection. The mice were sacrificed. (**B**) Metastatic cancer tissues were stained with H&E. (**C**) Vimentin expression in the CRC-derived liver metastases was detected by IHC. (**D**) Vimentin expression in tumors derived from LoVo cells was determined by qRT-PCR; ***P < 0.001. Scale bars, 100 μm in (**B**,**C**).

To test the role of RUFY3 cooperation with FOXK1 in the progression of CRC, we injected LoVo/EGFP-N1, LoVo/EGFP-RUFY3-scr shRNA and LoVo/EGFP-RUFY3-FOXK1 shRNA cells expressing green fluorescent protein (GFP) into nude mice to examine liver metastasis. Thirty days after injection, the mice with RUFY3 overexpressing LoVo cells, but not vector LoVo cells, formed a variety of large metastatic nodules in their livers (Fig. 6A), whereas the mice with FOXK1 knockdown in RUFY3-overexpressing cells had small liver nodules compared with those in the scr shRNA in RUFY3-overexpressing cells (Fig. 6A). The presence of liver metastases from CRC was confirmed by histological analysis (Fig. 6B).

To further demonstrate whether RUFY3 cooperation with FOXK1correlates with EMT, FOXK1 expression was repressed in RUFY3-overexpressing cells in orthotopic xenograft tumors. The overexpression of RUFY3 resulted in significant up-regulation of the mesenchymal marker vimentin in an IHC assay (Fig. 6C), whereas the loss of FOXK1 in RUFY3-overexpressed cells caused a decrease in vimentin expression, as determined by qRT-PCR (Fig. 6D).

Taken together, these results indicate that the RUFY3-FOXK1 axis has an important role in development and metastasis during CRC.

## Discussion

In this study, we characterized the role of RUFY3 in CRC cell growth, invasion and metastasis. Moreover, FOXK1 physically interacts with RUFY3, contributing to EMT, invasion and migration. Furthermore, patients with high co-expression of RUFY3 and FOXK1 had a shorter median survival than those without. Therefore, these findings suggest that the cooperative relationship between RUFY3 and FOXK1 plays a pivotal role in CRC. RUFY3 is highly expressed in brain tissue and has a role in neuronal development^12^. The N-terminal region of RUFY3 and its homologs, including RPIP8^27^ and RPIP9^28^, contain the RUN domain, which can interact with Rap2^27–29^ and Rab^10, 13^. Wang found significant upregulation of RUFY3 in gastric cancer samples compared with their non-tumor counterparts^14^. However, the molecular mechanisms and the role of RUFY3 in CRC remain unknown. Here, we have shown that overexpression of RUFY3 increased CRC cell proliferation and promoted colony formation in soft agar *in vitro*, whereas knockdown of RUFY3 expression by siRNA inhibited colony formation in soft agar, migration and invasion. These findings suggest that aberrant RUFY3 upregulation might be an important mechanism underlying tumor development in CRC cells.

Transcriptional factor FOXK1 is a member of the FOX family, which is involved in cell growth and metabolism. Higher FOXK1 expression causes a variety of diseases and may play an important role in the development of various tumors^17, 19, 30^. Protein-protein interaction analysis using the STRING database showed that RUFY3 and FOXK1 may have been some co-precipitation reactions, which has not been confirmed yet. We now report that both the protein level and cellular localization of RUFY3 were similar to FOXK1 in CRC; reciprocal co-immunoprecipitation assay showed that RUFY3 could bind FOXK1. These results indicate that the FOXK1-RUFY3 axis plays an important role in CRC.

EMT is an orchestration of physiomorphological events that lead to a change in the cell identity from epithelial to mesenchymal^19, 20, 23, 24^. During cancer progression, advanced tumor cells frequently exhibit a conspicuous downregulation of epithelial markers and loss of intercellular junctions, resulting in a loss of epithelial polarity and reduced intercellular adhesion. These alterations are often accompanied by increased cell motility and the expression of mesenchymal-specific proteins. A recent study demonstrated that IRGM1 enhances B16 cell migration and invasion via the induction of F-actin polymerization and EMT^31^. Consistently, we demonstrated that RUFY3 overexpression mediates actin polymerization, decreases E-cadherin expression and is paralleled by an increase in the mesenchymal marker vimentin. In contrast, FOXK1 downregulation in RUFY3-overexpressing cells was significantly associated with low expression levels of vimentin, but it was adversely associated with high E-cadherin expression. Similar results were obtained for these cells with RUFY3, which often exhibited a fibroblast-like, spindle-shaped phenotype, whereas FOXK1 knockdown in RUFY3 -overexpressing cells led to EMT reversion. Together, these results strongly suggested that RUFY3 and FOXK1 are mutually essential for maintaining EMT and metastatic phenotypes.

In conclusion, our results uncovered a novel, unexpected regulatory function of RUFY3. We identified FOXK1 as a new RUFY3-binding protein. This study shed light on the critical role of RUFY3 in inducing EMT and in the migration and invasion phenotypes caused by abnormal FOXK1 expression. Therefore, the RUFY3-FOXK1 axis might represent a novel target for treating CRC.

## Materials and Methods

### Reagents, cell cultures and cell lines

Cycloheximide (sc-3508), Rabbit anti-FOXK1 (H-140), E-cadherin (H-108) and mouse immunoglobulin G (nm IgG) were purchased from Santa Cruz (Santa-Cruz, CA, USA). Rabbit anti-Vimentin (Ag0489), MMP2 (Ag0549) and MMP9 (Ag0552), and mouse anti-human GAPDH were purchased from Proteintech (Wuhan, China). Rabbit anti-Erk1/2 (137F5), p- Erk1/2 (Thr202/Tyr204), Akt (C-terminal), and p-Akt (Ser473) were purchased from CST (CST, MA, USA). Anti-RIPX (Anti-RUFY3, ab89147) was purchased from Abcam (Cambridge, UK). LoVo and SW1116 cells were grown in RPMI 1640 containing 10% fetal bovine serum (Life Technologies, Monza, Italy), 1% glutamine (Life Technologies) and 1% penicillin/streptomycin (Life Technologies) in a humidified incubator at 37 °C with an atmosphere of 5% CO_2_.

### Ethics statement

The use of human CRC tissues were approved by the Medical Ethics Committee of Nanfang Hospital, Southern Medical University, China (NFEC-2017-026) and were performed in accordance with the approved guidelines. Informed consents were obtained from the patients.

All animal care and procedures were in accordance with China Nanfang Hospital policies for health and well-being. All animal experimental procedures were approved by Nanfang Hospital Animal Ethic Committee (NFYY-2016-15).

### Immunohistochemistry

Ninety-one surgically removed CRC samples from 2005 to 2008 were selected from the Department of Surgery of Nanfang Hospital, Southern Medical University. Paraffin-embedded tissue blocks were cut into 5 μm sections and transferred to glass slides. The slides were deparaffinized with xylene, rehydrated with ethanol, washed and subjected to microwave retrieval in a citrate buffer. Sections were then immersed in 3% hydrogen peroxide to block endogenous peroxidase activity and incubated with the primary antibodies, followed by incubation with peroxidase-conjugated anti-rabbit secondary antibody (Dako) (1:100). The expression levels of FOXK1 and RUFY3 were then visualized using 1 mg/ml 3,3′-diaminobenzidine and counterstained with hematoxylin. Normal mouse IgG (Sigma) was used as an isotype control for anti-FOXK1 or RUFY3 antibody to verify the staining specificity. Histopathological analyses confirmed the malignant tissues. Tumor staging was defined according to the criteria for histological classification proposed by the International Union against Cancer (UICC). Tissues in which more than 10% of the cancer cells were positively stained were considered positive. For the quantitative analysis, the ratio of positively stained cells to tumor cells in five random areas at 200-fold magnification was recorded. The tissue slides were independently scored by two investigators; intensity of staining of cancer cells was scored as 0 (no staining), 1 (weak staining, light yellow), 2 (moderate staining, yellowish brown) and 3 (strong staining, brown). An intensity score of ≥2 with at least 50% of positive cells was considered as high expression (or overexpression), and <50% of positive cells or <2 in intensity score was regarded as low expression. The study was approved by the institutional human ethics committees of the relevant institutions.

### Constructs and establishment of stable transfectants

Normal human complementary DNA (cDNA) corresponding to the full-length RUFY3 was obtained by RT-PCR amplification. The PCR aliquots were subcloned into mammalian expression vector pENTER-FLAG (ViGene Biosciences, Rockville, MD, USA). Populations of pENTER vector alone and pENTER RUFY3 stable transfectants were obtained using the same plasmid and selection process as previously described^19^. Viable clones were pooled, identified for RUFY3 expression by western blot and maintained in medium containing 600 μg/ml puromycin for additional studies.

### RNA isolation and quantitative real-time RT-PCR

Cells were harvested, and total RNA was extracted using TRIzol Reagent (Gibco BRL, Gaithersburg, MD). RNA was reverse transcribed to cDNA with Thermoscript RT system reagent (Gibco BRL) in accordance with the manufacturer’s instructions.

Quantitative real-time PCR was performed using an Applied Biosystems Sequence Detection System 7900 (ABI Prism 7900HT, Applied Biosystems Company, USA) with a 10-µl mixture consisting of Power SYBR GREEN PCR Master Mix (Applied Biosystems, Foster City, CA), 500 nmol of each primer, and 300 ng of cDNA templates. The reactions were performed with an initial denaturation at 95 °C for 5 minutes, which was followed by 60 cycles of 20 seconds at 94 °C, 20 seconds at 60 °C, and 40 seconds at 72 °C. A final extension at 72 °C for 5 minutes was included before a temperature ramp from 72 °C to 95 °C at 0.1 °C/s with continuous fluorescent acquisition. Each cDNA sample was duplicated for each q-RT-PCR attempt, and the average relative fold mRNA expression levels were determined using the 2^−ΔΔCt^ method; GAPDH was the internal control. The oligonucleotide primers for FOXK1, SNAI1, SNAI2, KLF8, Sp1, Sp3, YY1, HMGA1 and GAPDH mRNA are listed in Supplementary Table 1.

### Gene silencing using siRNA

The sequences of the FOXK1 siRNA, RUFY3 siRNA and Scrambled (Scr) siRNA were CCAUCAAGAUCCAGUUCAC dTdT, UCUCAAGCAUGAACUUGCCUUUAAG and CGUACGCGGAAUACUUCGA, respectively, as previously described in detail^14, 19^. The siRNA transfection was performed using Lipofectamine 2000 (Invitrogen, USA) according to the manufacturer’s instructions.

### Western blot analysis and immunofluorescence

For western blot analysis, cells were harvested and lysed in lysis buffer. A total of 30 μg of protein lysates was separated by SDS-PAGE and transferred onto a PVDF membrane. Primary antibodies were diluted according to the company’s recommendation. Protein bands were detected using ECL Western Blotting Detection Reagent (GE Healthcare).

Cells grown on cover glass were fixed with 4% paraformaldehyde, and nonspecific binding was blocked by incubation with 1% bovine serum albumin. The glasses were probed with the primary antibodies prior to incubation with TR- or fluorescein isothiocyanate-conjugated second antibodies. After mounting, the slips were visualized under an Olympus CKX 41 fluorescence microscope (Olympus Optical Co., Ltd., Tokyo, Japan) or a Zeiss LSM710 confocal microscope using the 40x or 63x objectives (ZEISS International, Germany).

For staining of F-actin, cells were washed with PBS, fixed in methanol/acetone (1:1) for 5 min on ice, and incubated with rhodamine-conjugated phallotoxin (5 U/mL, Molecular Probes) in PBS at a 1:40 dilution for 1 h. Coverslips were washed, mounted, and visualized using fluorescence microscope. Nuclei were stained with 1 μg/mL Hoechst 33258, and cells were analyzed using a fluorescence microscope.

### Soft agar and WST-1 cell proliferation assays

The pooled stable transfectants of vector and RUFY3, or scrambled siRNA (scr-siRNA) and RUFY3-siRNA cell, were plated in triplicate on plates containing 0.35% agar on top of a 0.7% agar base. Colonies were scored after staining with Coomassie Brilliant Blue R250. Only those colonies containing at least 50 cells were considered viable. The Cell Proliferation Reagent WST-1 was a ready-to-use colorimetric assay (Roche Diagnostics).

### Cell migration and invasion assays

Invasion and cell migration assays were performed as follows, cells were plated in serum-free medium on Transwell inserts (Corning, NY) coated with 25 μg of Matrigel (BD Biosciences) for invasion assays. After incubation for 48 h at 37 °C/5% CO_2_, the inserts were fixed with 3.7% paraformaldehyde/PBS and stained with 2% crystal violet. The number of cells that had invaded was counted in five representative (×200) fields per insert. Cell migration assays were performed. Briefly, cells plated in six-well plates with 100% confluence were wounded with a pipette tip at time 0. The medium was changed to remove cell debris, and the cells were cultured in the presence of 10 μg/ml mitomycin C to inhibit cell proliferation. Photographs were taken 60 h later.

### Co-Immunoprecipitation (Co-IP)

To precipitate the target proteins, the lysates of cells with or without pooled stable transfection of tagged constructs were incubated with 3 µg of the primary antibody for 3 h at 4 °C, which was followed by incubation with a precleared protein A-agarose bead (Roche, Mannheim, Germany) slurry. After extensive washing, samples were subjected to western blot analysis to detect potentially interacting proteins.

### Construction and production of recombinant lentivirus

The RUFY3-overexpressing cell line (EGFP + RUFY3 (+)) was constructed using the lentivirus expressing RUFY3 cDNA (Shanghai Genechem, China), and an EGFP-lentiviral vector was used as a negative control. Clone identity was verified by sequencing. To establish the RUFY3 overexpressing and FOXK1 knockdown (EGFP-RUFY3-FOXK1 shRNA) cell lines, lentiviruses expressing RUFY3 cDNA and FOXK1 shRNA or scrambled (Scr) shRNA (Supplementary Table 1) were co-transfected into the LoVo cell line. All lentiviruses were then mixed with 5 μg/mL polybrene to enhance the transfection efficiency.

### *In vivo* assays for tumor growth and metastasis

To evaluate the metastatic potential of cancer cells to the liver *in vivo*, 5 × 10^6^ LoVo/EGFP-N1, LoVo/EGFP-RUFY3-scr shRNA and LoVo/EGFP-RUFY3-FOXK1 shRNA cells were injected into the spleens of orthotopic of mice (n = 3 for each group). One month later, the mice were sacrificed; the individual organs were removed and assessed using the *In Vivo* F Imaging System (Kodak). The metastatic tissues were analyzed with H&E, IHC and qRT-PCR.

### Statistical analysis

Statistical analysis was performed using the SPSS statistical software package (standard version 13.0; SPSS, Chicago, IL). The quantitative data that we obtained from experiments with biological replicates are shown as the means (±SD). Means from each group were compared using t-tests. Linear regression and Pearson Correlation Analysis were performed to assess the relationship between RUFY3 and FOXK1 in the tissue. Probability values from the two-tailed test of less than 0.05 were considered significant.

## Electronic supplementary material

supplementary information

## References

[1] Mari M, Macia E, Le Marchand-Brustel Y, Cormont M (2001). Role of the FYVE finger and the RUN domain for the subcellular localization of Rabip4. J Biol Chem.

[2] Yang J, Kim O, Wu J, Qiu Y (2002). Interaction between tyrosine kinase Etk and a RUN domain- and FYVE domain-containing protein RUFY1. A possible role of ETK in regulation of vesicle trafficking. J Biol Chem.

[3] Cormont M, Mari M, Galmiche A, Hofman P, Le Marchand-Brustel Y (2001). A FYVE-finger-containing protein, Rabip4, is a Rab4 effector involved in early endosomal traffic. Proc Natl Acad Sci USA.

[4] Dunkelberg JC, Gutierrez-Hartmann A (2001). LZ-FYVE: a novel developmental stage-specific leucine zipper, FYVE-finger protein. DNA Cell Biol.

[5] Wei Z, Sun M, Liu X, Zhang J, Jin Y (2014). RUFY3, a protein specifically expressed in neurons, interacts with actin-bundling protein Fascin to control the growth of axons. J Neurochem.

[6] Terawaki S (2015). RUN and FYVE domain-containing protein 4 enhances autophagy and lysosome tethering in response to Interleukin-4. J Cell Biol.

[7] Vukmirica J, Monzo P, Le Marchand-Brustel Y, Cormont M (2006). The Rab4A effector protein Rabip4 is involved in migration of NIH 3T3 fibroblasts. J Biol Chem.

[8] Ivan V (2012). AP-3 and Rabip4’ coordinately regulate spatial distribution of lysosomes. PLoS One.

[9] He J (2009). Membrane insertion of the FYVE domain is modulated by pH. Proteins.

[10] Fukuda M, Kobayashi H, Ishibashi K, Ohbayashi N (2011). Genome-wide investigation of the Rab binding activity of RUN domains: development of a novel tool that specifically traps GTP-Rab35. Cell Struct Funct.

[11] Yamamoto H (2010). Functional cross-talk between Rab14 and Rab4 through a dual effector, RUFY1/Rabip4. Mol Biol Cell.

[12] Mori T, Wada T, Suzuki T, Kubota Y, Inagaki N (2007). Singar1, a novel RUN domain-containing protein, suppresses formation of surplus axons for neuronal polarity. J Biol Chem.

[13] Yoshida H, Okumura N, Kitagishi Y, Shirafuji N, Matsuda S (2010). Rab5(Q79L) interacts with the carboxyl terminus of RUFY3. Int J Biol Sci.

[14] Wang G (2015). PAK1 regulates RUFY3-mediated gastric cancer cell migration and invasion. Cell Death Dis.

[15] Garry DJ, Yang Q, Bassel-Duby R, Williams RS (1997). Persistent expression of MNF identifies myogenic stem cells in postnatal muscles. Dev Biol.

[16] Garry DJ (2000). Myogenic stem cell function is impaired in mice lacking the forkhead/winged helix protein MNF. Proc Natl Acad Sci U S A.

[17] Yang Q (2000). The winged-helix/forkhead protein myocyte nuclear factor beta (MNF-beta) forms a co-repressor complex with mammalian sin3B. Biochem J.

[18] Wang W (2015). FOXKs promote Wnt/β-catenin signaling by translocating DVL into the nucleus. Dev Cell.

[19] Wu, Y. *et al*. Oncogene FOXK1 enhances invasion of colorectal carcinoma by inducing epithelial-mesenchymal transition. *Oncotarget*. doi:10.18632/oncotarget.9457 (2016). 10.18632/oncotarget.9457 PMC5239465 27223064

[20] Peng, Y. *et al*. Direct regulation of FOXK1 by c-jun promotes proliferation, invasion and metastasis in gastric cancer cells. *Cell death Disease* (2016). 10.1038/cddis.2016.225 PMC5260906 27882939

[21] Shi X, Seldin DC, Garry DJ (2012). Foxk1 recruits the Sds3 complex and represses gene expression in myogenic progenitors. Biochem J..

[22] Garry DJ (2000). Myogenic stem cell function is impaired in mice lacking the forkhead/winged helix protein MNF. Proc Natl Acad Sci USA.

[23] Zhang W (2011). Four and a half LIM protein 2 (FHL2) negatively regulates the transcription of E-cadherin through interaction with Snail1. Eur J Cancer.

[24] Yan Q (2015). KLF8 promotes tumorigenesis, invasion and metastasis of colorectal cancer cells by transcriptional activation of FHL2. Oncotarget.

[25] Yang MH (2008). Direct regulation of TWIST by HIF-1alpha promotes metastasis. Nat Cell Biol.

[26] Okada T (2015). The Rho GTPase Rnd1 suppresses mammary tumorigenesis and EMT by restraining Ras-MAPK signalling. Nat Cell Biol.

[27] Janoueix-Lerosey I, Pasheva E, de Tand MF, Tavitian A, de Gunzburg J (1998). Identification of a specific effector of the small GTP-binding protein Rap2. Eur J Biochem.

[28] Wang S (2003). Cloning, expression, and genomic structure of a novel human Rap2 interacting gene (RPIP9). Biochem Genet.

[29] Nancy V (1999). Identification and characterization of potential effector molecules of the Ras-related GTPase Rap2. J Biol Chem.

[30] Bowman CJ, Ayer DE, Dynlacht BD (2014). Foxk proteins repress the initiation of starvation-induced atrophy and autophagy programs. Nat Cell Biol.

[31] Tian L (2015). IRGM1 enhances B16 melanoma cell metastasis through PI3K-Rac1 mediated epithelial mesenchymal transition. Sci Rep..

