# Supplementary material for: RUFY3 interaction with FOXK1 promotes invasion and metastasis in colorectal cancer
Source: Sci Rep. 2017 Jun 16;7:3709. doi: 10.1038/s41598-017-04011-1 (PMC5473929; doi:10.1038/s41598-017-04011-1)
Supplement: Supplementary file 1 — supplementary information [file 41598_2017_4011_MOESM1_ESM.doc]

**Rufy3 interaction with FOXK1 promotes invasion and metastasis in colorectal cancer**

Ruyi Xie^1^, Jing Wang^1^, Xuehua Liu^1,2^, Liqing Wu^1^, Hui Zhang^1,3^, Weimei Tang^1^, Yueqiao Li^1^, Li Xiang^1,4^, Ying Peng^1^, Xiaoting Huang^1^, Yang Bai^1^, Guangnan Liu^1^, Aimin Li^1^, Yadong Wang^1^, Ye Chen^1^, Yuexin Ren^1^, Guoxin Li^5^, Wei Gong^1^, Side Liu^1#^, Jide Wang^1#^

**Supplementary Information**

**
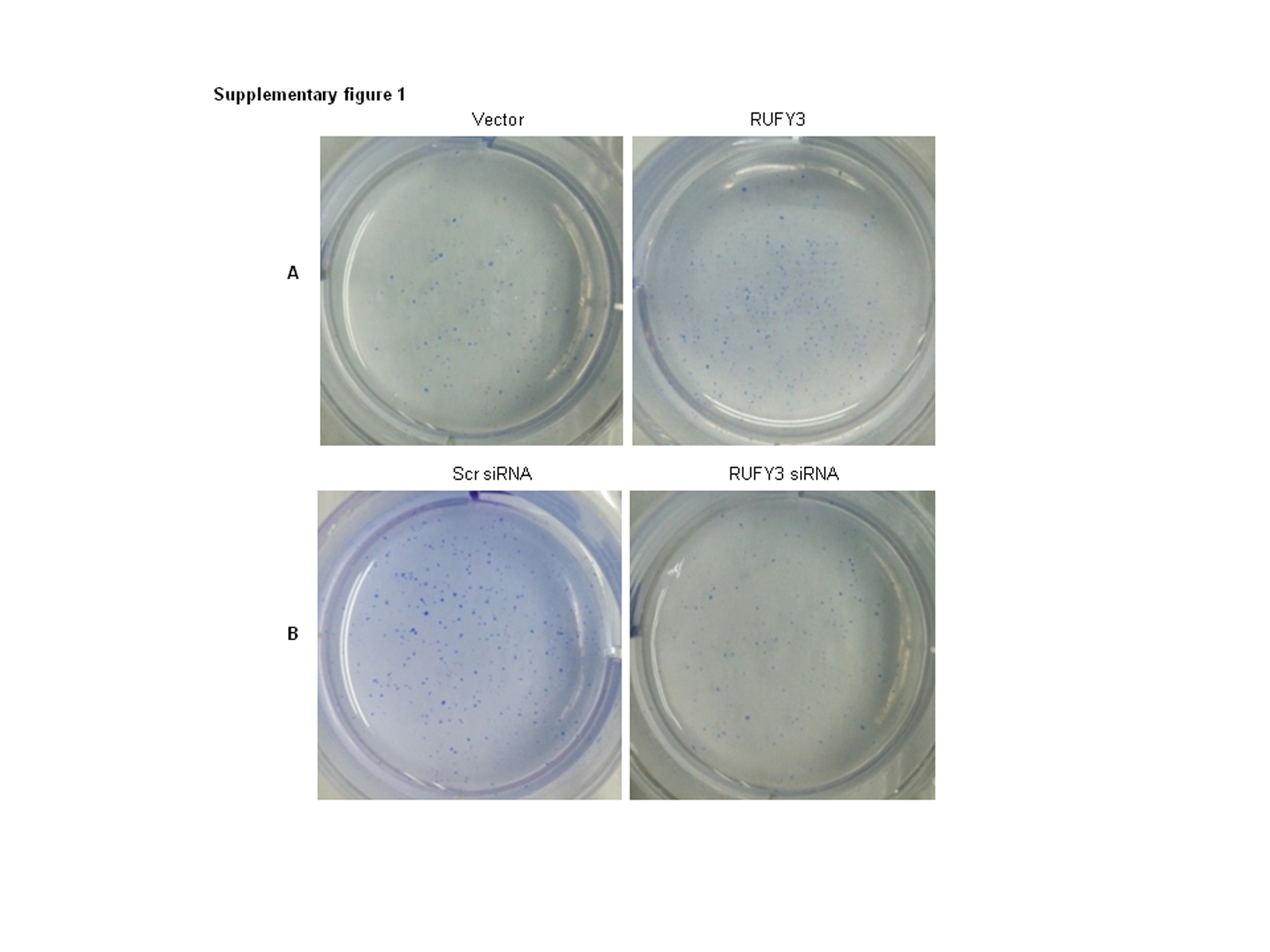
**

**Supplementary figure 1. RUFY3 regulated anchorage-dependent and anchorage- independent cell growth.** (**A & B**) Vector and pooled stable RUFY3 transfectants, Scr siRNA and RUFY3 siRNA in LoVo cells and their corresponding control cells were plated in tissue culture dishes with complete culture medium containing 0.35% agar on the top and 0.5% agar on the bottom. After 12 days, cell colonies were visualized by staining with 0.005% crystal violet.


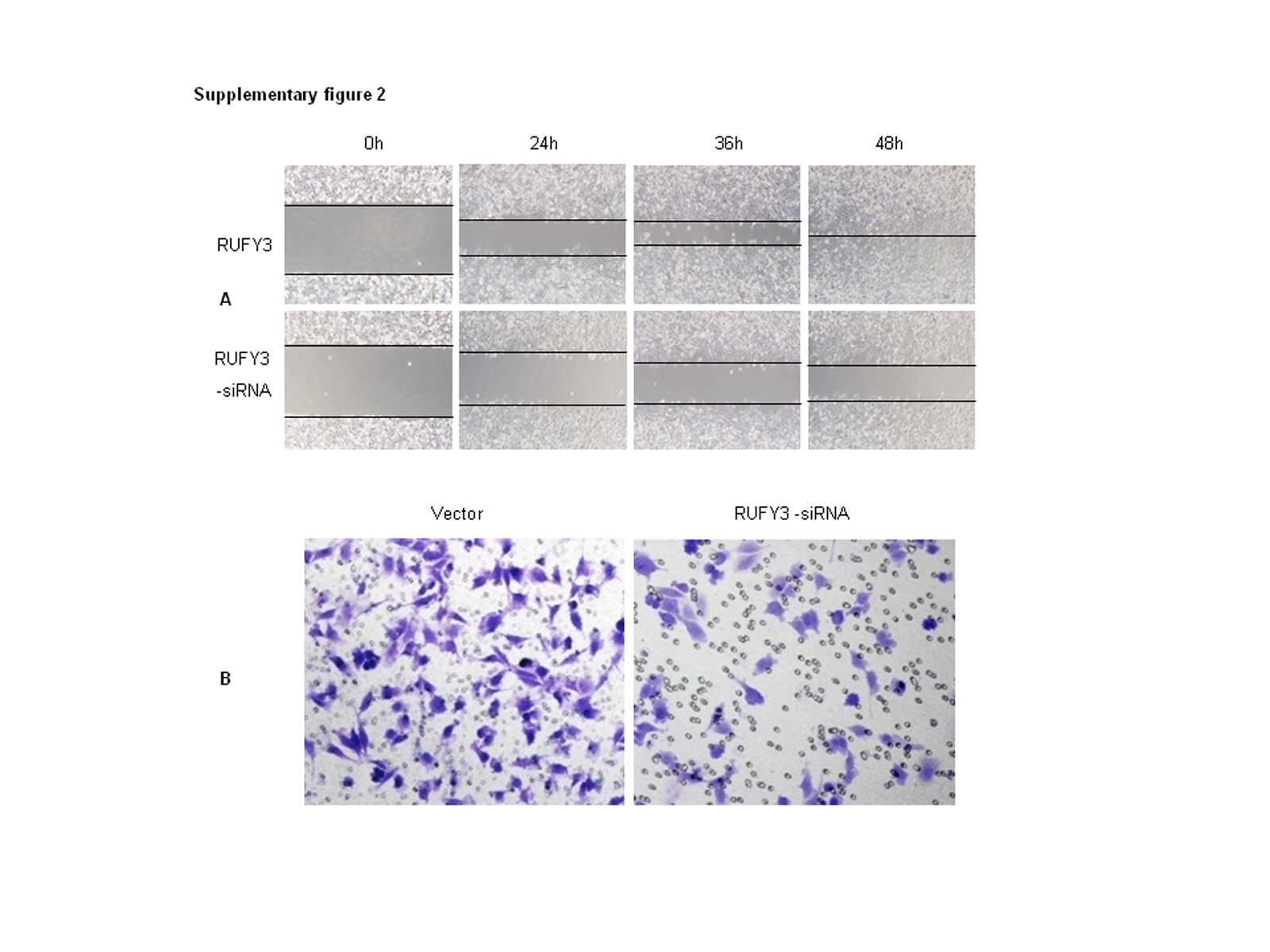


**Supplementary figure 2. Knockdown of RUFY3 led to a significant inhibition of the migratory and invasive ability of CRC cells**. **(A)** Knockdown of RUFY3 led to a significantly slower migration at 24, 36 and 48 h after cells were infected with RUFY3 siRNA. **(B)** Inhibition of RUFY3 led to a reduced invasive ability of LoVo cells. The experiments were repeated at least three times.

**
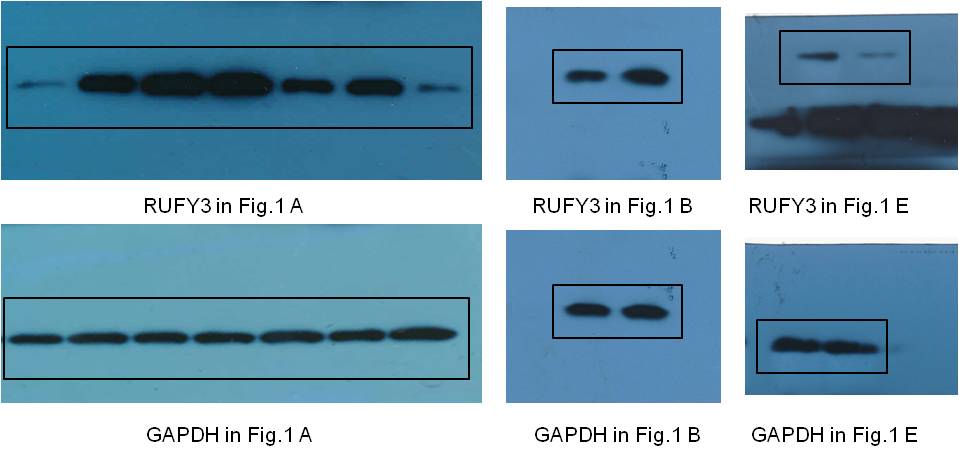
**

**Supplementary figure 3**. The full-length blots/gels including the key data presented in Fig. 1.


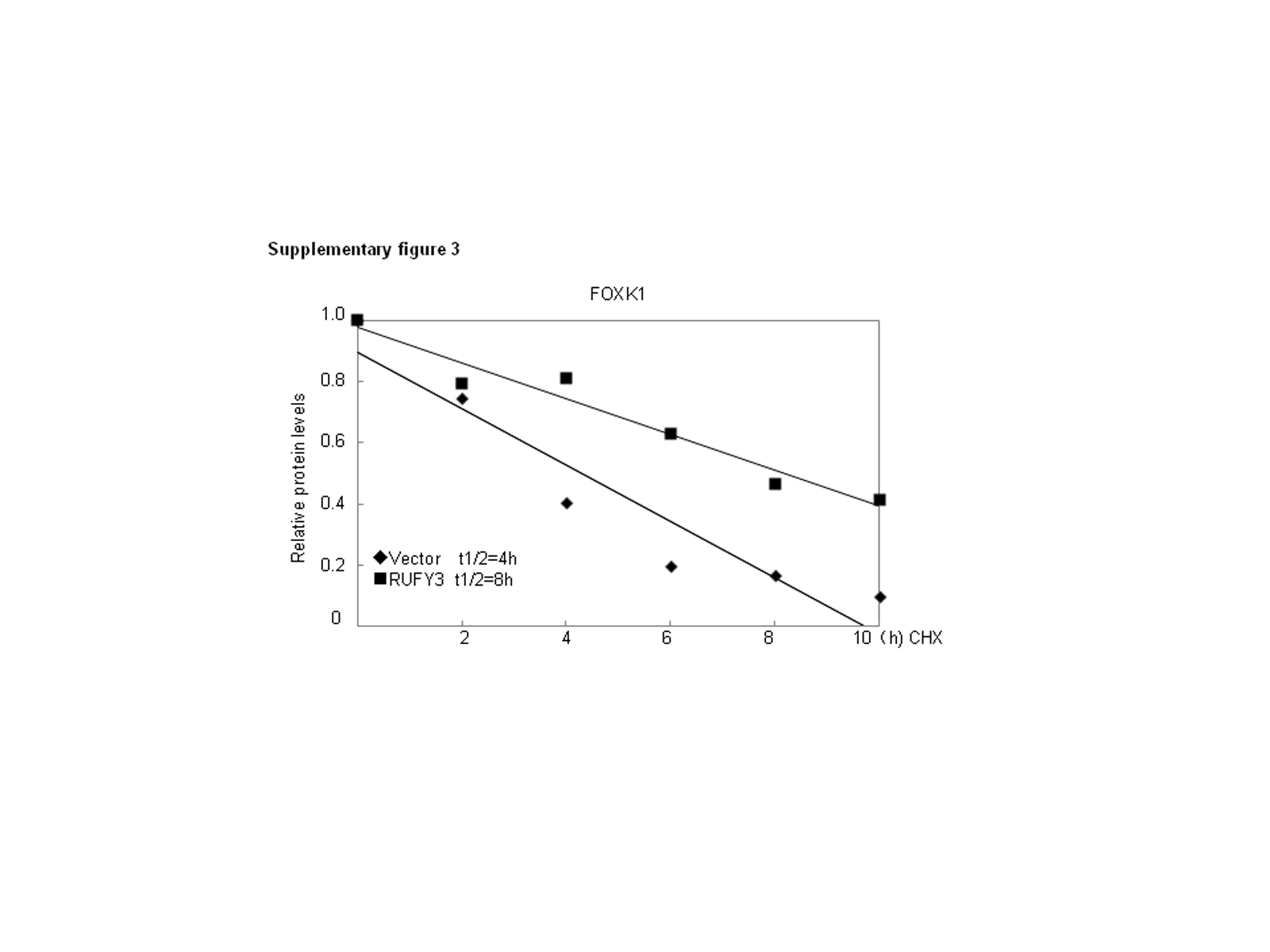


**Supplementary figure 4.** Regulation of FOXK1 protein stability by RUFY3. A quantitation of Western blotting measuring FOXK1 stability in the indicated cells. The values are normalized to the expression of GAPDH.


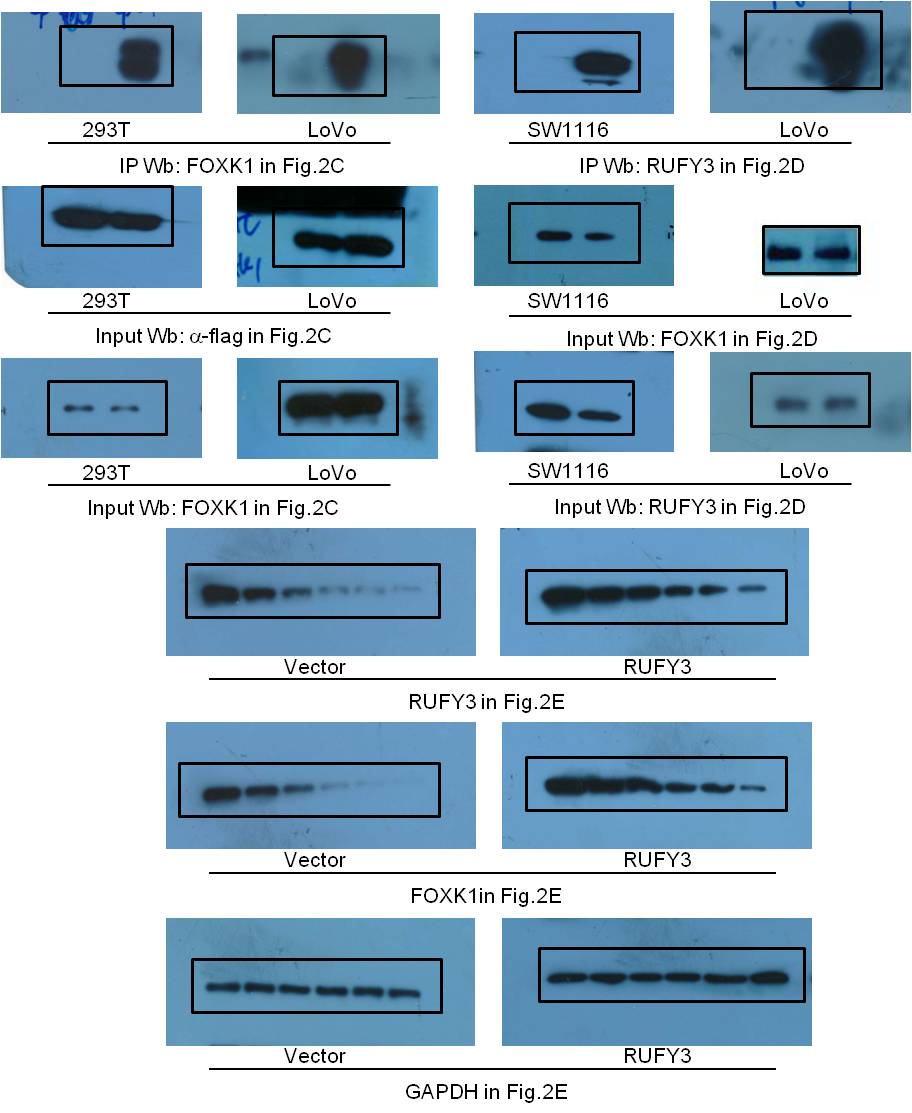


**Supplementary figure 5**. The full-length blots/gels including the key data presented in Fig. 2.


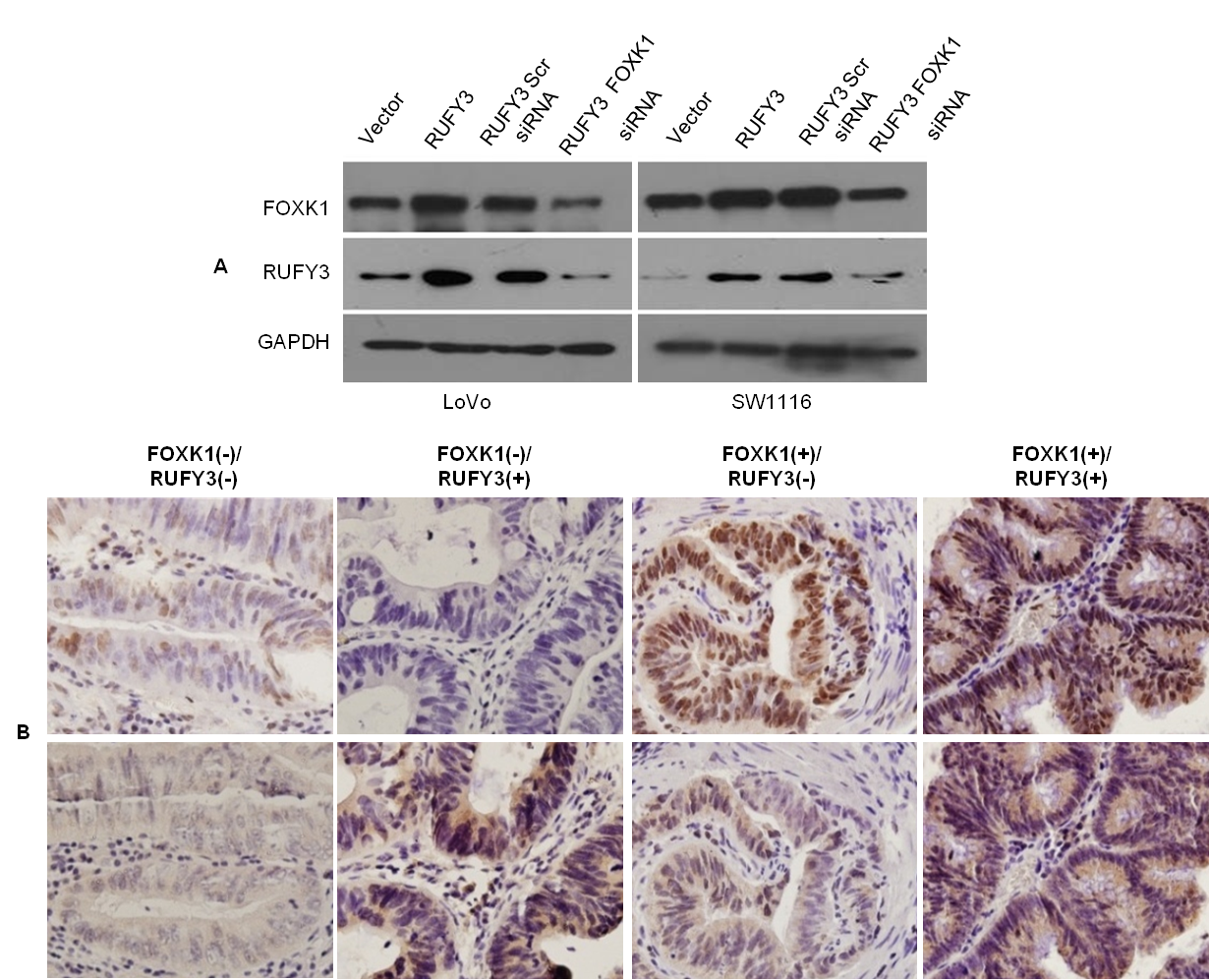


**Supplementary figure 6. RUFY3 and FOXK1 are co-expressed in primary CRC. (A)** CRC cell pooled stable transfectants of Vector or RUFY3, by transfection with FOXK1 siRNA or Scr siRNA for 48 h. The protein levels of RUFY3 and FOXK1 were detected using western blot analysis. (**B)** Representative IHC images for tissues are shown. Scale bars represent 100 μm in **B**.

**
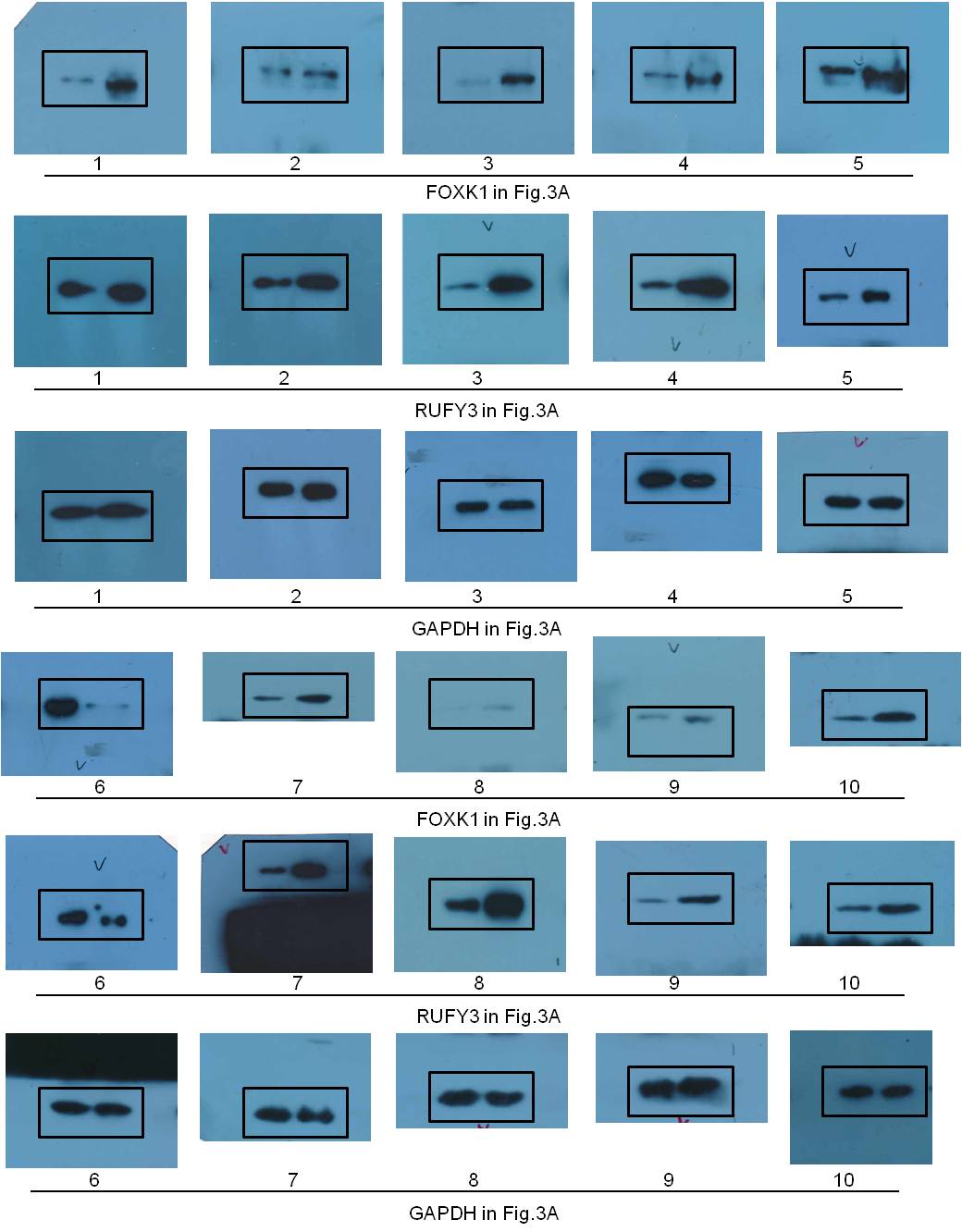
**

**Supplementary figure 7**. The full-length blots/gels including the key data presented in Fig. 3.


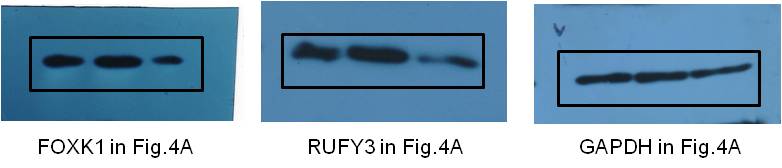


**Supplementary figure 8**. The full-length blots/gels including the key data presented in Fig. 4.

**
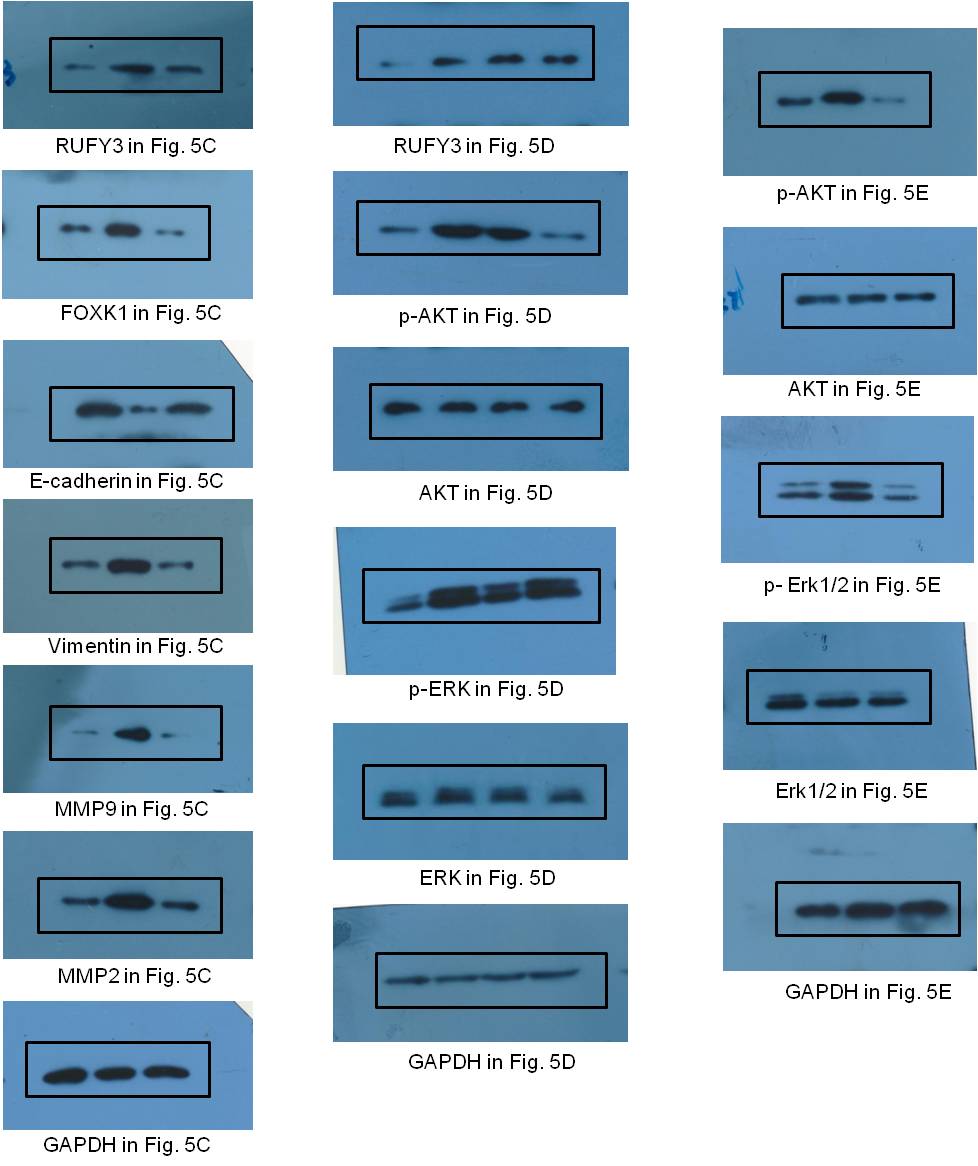
**

**Supplementary figure 9**. The full-length blots/gels including the key data presented in Fig. 5.

Supplementary Table 1. Oligonucleotides Sequences

| Application | Gene | Sequence |
| --- | --- | --- |
| qPCR | FOXK1 | F: 5’- GCAGTGTACCTTCCGGTTTC -3’ |
|  |  | R: 5’- AGGAGTTGGGGACACTGATG -3’ |
|  | SNAI1 | F: 5’- TTTACCTTCCAGCAGCCCTA -3’ |
|  |  | R: 5’- CCCACTGTCCTCATCTGACA -3’ |
|  | SNAI2 | F: 5’- GAGCATACAGCCCCATCACT -3’ |
|  |  | R: 5’- GGGTCTGAAAGCTTGGACTG -3’ |
|  | KLF8 | F: 5’- TATCTCCTCGTGGGTTCCTG -3’ |
|  |  | R: 5’- TCCGTTCCCCTTTTACCTCT -3’ |
|  | Sp1 | F: 5’- CTGGTGGGCAGTATGTTGTG -3’ |
|  |  | R: 5’- TTGGTTTGCACCTGGTATGA -3’ |
|  | Sp3 | F: 5’- GCTCCACCTTTTGTGTTTCC -3’ |
|  |  | R: 5’- TCTTGTTTCACGGGCTTTTC -3’ |
|  | YY1 | F: 5’- ACGGCTTCGAGGATCAGATT -3’ |
|  |  | R: 5’- GCCGCTGAGGTAACTCTTCTT -3’ |
|  | HMGA1 | F: 5’- GAAAAGGACGGCACTGAGAA -3’ |
|  |  | R: 5’- CTTCCTGGAGTTGTGGTGGT -3’ |
|  | GAPDH | F: 5’- GTCAACGGATTTGGTCGTATT- 3’ |
|  |  | R: 5’- CTCCTGGAAGATGGTGATGGG -3’ |
| FOXK1-shRNA | | CCGGGAGACAGCCCCAAGGATGATCAAGAGTCATCCTTGGGGCTGTCTCTTTTTG |

**Supplementary Table 2. Correlation between RUFY3/FOXK1 protein expression and the clinicopathological parameters of colorectal carc**inoma

| Features | Total number（n=91） | | | Expression of RUFY3 | |  | | Expression of FOXK1 | |  |
| --- | --- | --- | --- | --- | --- | --- | --- | --- | --- | --- |
|  |  |  |  | Low (%) | High (%) | P values | | Low (%) | High (%) | P values |
| Age(years) | | | | | | | |  |  |  |
| <60 | 18 | | | 6 (33.3) | 12 (66.7) | 0.554 | | 6(33.3) | 12(66.7) | 0.545 |
| ≥60 | 73 | | | 26 (35.6) | 47 (64.4) |  |  | 23(31.5) | 50(68.7) |  |
| Gender | | | | | | | |  |  |  |
| Male | 46 | | | 15 (32.6) | 31 (67.4) | 0.383 | | 16(34.8) | 30(65.2) | 0.353 |
| Female | 45 | | | 17 (37.8) | 28 (62.2) |  |  | 13(28.9) | 32(71.1) |  |
| Differentiation | | | | | | | |  |  |  |
| Well | | 11 | | 7 (63.6) | 4 (36.4) | 0.018 | | 8(72.7) | 3(27.3) | 0.00 |
| Moderate | | 63 | | 23 (36.5) | 40 (63.5) |  |  | 21(33.3) | 42(66.7) |  |
| Poor | | 17 | | 2 (11.8) | 15 (88.2) |  |  | 0(0) | 17(100) |  |
| AJCC stage | | | | | | | |  |  |  |
| I ,II | 55 | | | 25 (45.5) | 30(54.5) | 0.009 | | 22(40.0) | 33(60.0) | 0.032 |
| III, IV | 36 | | | 7 (19.4) | 29 (80.6) |  |  | 7(19.4) | 29(80.6) |  |
| Location | | | | | | | |  |  |  |
| C,A,T | 44 | | | 14 (31.8) | 30 (68.2) | 0.335 | | 14(31.8) | 30(68.2) | 0.585 |
| D,S,R | 47 | | | 18 (38.3) | 29 (61.7) |  |  | 15(31.9) | 32(68.1) |  |
| Lymph node metastasis | | | | | | | |  |  |  |
| No | 56 | | | 25 (44.6) | 31 (55.4) | 0.014 | | 22(39.3) | 34(60.7) | 0.044 |
| Yes | 35 | | | 7 (20.0) | 28 (80.0) |  |  | 7(20.0) | 28(80.0) |  |
| Tumor size | | | | | | | |  |  |  |
| <3 | 5 | | | 3 (60.0) | 2 (40.0) | 0.232 | | 3 (60.0) | 2 (40.0) | 0.183 |
| ≥3 | 86 | | | 29 (33.7) | 57 (66.3) |  |  | 26(30.2) | 60(69.8) |  |
| TNM stage | | | | | | | |  |  |  |
| Tis,T1,T2 | | 6 | | 5 (83.3) | 1 (16.7) | 0.019 | | 5 (83.3) | 1 (16.7) | 0.012 |
| T3,T4 | | 85 | | 27 (31.8) | 58 (68.2) |  |  | 24(28.2) | 61(68.1) |  |
| Serosal invasion | | | | | | | |  |  |  |
| No | 6 | | | 5(83.3) | 1(16.7) | 0.019 | 5(83.3) | | 1(16.7) | 0.012 |
| Yes | 85 | | | 27(31.8) | 58(68.2) |  | 24(28.2) | | 61(71.8) |  |
| Expression of FOXK1 | | | | | | | |  |  |  |
| Low expression | | | 29 | 19(65.5) | 10(34.5) | 0.000 |  | |  |  |
| High expression | | | 62 | 13(21.0) | 49(79.0) |  |  | |  |  |
